# Tris(3-methyl­anilinium) penta­chlorido­anti­monate(III) chloride

**DOI:** 10.1107/S1600536811049087

**Published:** 2011-11-23

**Authors:** Ming-Liang Liu

**Affiliations:** a College of Chemistry and Chemical Engineering, Southeast University, Nanjing 211189, People’s Republic of China

## Abstract

In the title compound, (C_7_H_10_N)_3_[SbCl_5_]Cl, the Sb^III^ cation is coordinated by five Cl^−^ anions in a distorted square-pyramidal geometry, in which the longest Sb—Cl distance of 3.0319 (14) Å indicates a weak coordination bond. In the crystal, the 3-methyl­anilinium cations link with the complex antimonate anions and Cl^−^ anions *via* N—H⋯Cl hydrogen bonds.

## Related literature

For background to the title compound, see: Fu *et al.* (2011 ▶); Zhang *et al.* (2010 ▶). For related structures, see: Chen (2009*a* ▶,*b* ▶); Vijjulatha *et al.* (1997 ▶); Wei *et al.* (2008 ▶); Zhai *et al.* (2007 ▶).
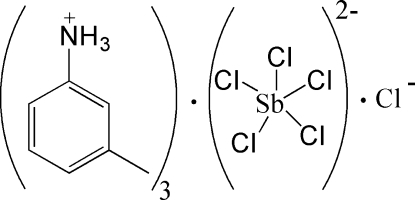


## Experimental

### 

#### Crystal data

(C_7_H_10_N)_3_[SbCl_5_]Cl
*M* *_r_* = 658.93
Monoclinic, 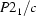

*a* = 17.171 (3) Å
*b* = 9.4065 (19) Å
*c* = 20.958 (8) Å
β = 122.36 (2)°
*V* = 2859.4 (13) Å^3^
*Z* = 4
Mo *K*α radiation
μ = 1.54 mm^−1^
*T* = 293 K
0.36 × 0.32 × 0.28 mm

#### Data collection

Rigaku SCXmini diffractometer
Absorption correction: multi-scan (*CrystalClear*; Rigaku, 2005 ▶) *T*_min_ = 0.566, *T*_max_ = 0.640
23714 measured reflections
5029 independent reflections
3776 reflections with *I* > 2σ(*I*)
*R*_int_ = 0.061

#### Refinement

*R*[*F* ^2^ > 2σ(*F* ^2^)] = 0.045
*wR*(*F* ^2^) = 0.085
*S* = 1.07
5029 reflections
286 parameters
H-atom parameters constrained
Δρ_max_ = 0.45 e Å^−3^
Δρ_min_ = −0.57 e Å^−3^

### 

Data collection: *CrystalClear* (Rigaku, 2005 ▶); cell refinement: *CrystalClear*; data reduction: *CrystalClear*; program(s) used to solve structure: *SHELXTL* (Sheldrick, 2008 ▶); program(s) used to refine structure: *SHELXTL*; molecular graphics: *SHELXTL*; software used to prepare material for publication: *SHELXTL*.

## Supplementary Material

Crystal structure: contains datablock(s) I, global. DOI: 10.1107/S1600536811049087/xu5391sup1.cif

Structure factors: contains datablock(s) I. DOI: 10.1107/S1600536811049087/xu5391Isup2.hkl

Additional supplementary materials:  crystallographic information; 3D view; checkCIF report

## Figures and Tables

**Table 1:** Selected bond lengths (Å)

Sb1—Cl2	3.0319 (14)
Sb1—Cl3	2.5325 (14)
Sb1—Cl4	2.4182 (15)
Sb1—Cl5	2.4043 (13)
Sb1—Cl6	2.7779 (14)

**Table 2:** Hydrogen-bond geometry (Å, °)

*D*—H⋯*A*	*D*—H	H⋯*A*	*D*⋯*A*	*D*—H⋯*A*
N1—H1*D*⋯Cl1	0.89	2.72	3.602 (4)	169
N1—H1*E*⋯Cl6^i^	0.89	2.67	3.513 (4)	158
N1—H1*F*⋯Cl2^ii^	0.89	2.59	3.455 (4)	163
N2—H2*A*⋯Cl1	0.89	2.37	3.240 (4)	166
N2—H2*B*⋯Cl1^iii^	0.89	2.40	3.249 (4)	160
N2—H2*C*⋯Cl2^iv^	0.89	2.41	3.238 (4)	155
N3—H3*A*⋯Cl1^v^	0.89	2.47	3.344 (5)	167
N3—H3*B*⋯Cl2^iii^	0.89	2.56	3.366 (5)	151
N3—H3*C*⋯Cl1^iii^	0.89	2.55	3.355 (5)	151

Symmetry codes: (i) 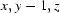; (ii) 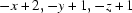; (iii) 
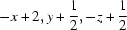
; (iv) 
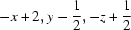
; (v) 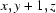.

**Fig. 1.**
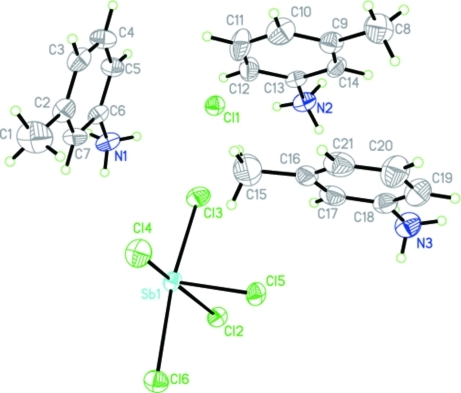
The molecular structure of the title compound, showing the atomic numbering scheme with 30% probability displacement ellipsoids.

**Fig. 2.**
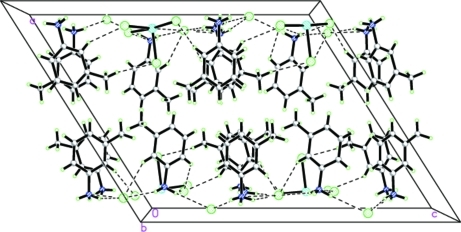
Crystal structure of the title compound with view along the b axis. Intermolecular interactions are shown as dashed lines.

## supplementary crystallographic information

### Comment

Recently much attention has been devoted to crystals containing organic ions and inorganic ions due to the tunebility of their special structural features and their potential ferroelectrics property (Fu *et al.*, 2011; Zhang *et al.*, 2010). In our laboratory, the title compound has been synthesized and its crystal structure is herein reported.

The title compound, [(C_7_H_10_N)_3_SbCl_5_]^+^.Cl^-^, has an asymmetric unit that consists of three C_7_H_10_N cations, one antimony(III) pentachloride anion and one chloride anion all in general positions (Fig 1). The non-hydrgen atoms of C_7_H_10_N cation are nearly coplanar, the antimony(III) atom is coordinated by five chlorine atoms, forming a distorted square-pyramid, the Sb—Cl bond distances range from 2.4043 (13) to 3.0319 (14) Å (Table 1). This range of values is compared to those observed in dimorpholinium pentachloridoantimonate(III) (2.045 (8)–2.92230 (9) Å; Chen *et al.*, 2009*a*) and that reported for diisonicotinium pentachloridoantimonate(III) monohydrate (2.3642 (12) to 2.9002 (14) Å; Chen *et al.*, 2009*b*). The existence of N—H···Cl hydrogen-bonding interactions gives rise a three-dimensional structure (Fig. 2).

### Experimental

3.21 g (0.03 mol) of 3-methylbenzenamine was firstly dissolved in 30 ml ethanol, to which 1.1 g (0.03 mol) of hydrochloric acid was then added to afford the solution, then the 2.28 g (0.01 mol) antimony chloride was dissolved in 20 ml ethanol which was added hydrochloric acid, at last, mixed the above solution without any precipitation under stirring at the ambient temperature. Single crystals suitable for X-ray structure analysis were obtained by the slow evaporation of the above solution after 4 days in air.

The dielectric constant of the compound as a function of temperature indicates that the permittivity is basically temperature-independent (*ε* = C/(T–T_0_)), suggesting that this compound is not ferroelectric or there may be no distinct phase transition occurring within the measured temperature within the measured temperature (below the melting point).

### Refinement

H atoms were placed in calculated positions (N—H = 0.89 Å; C—H = 0.93–0.97 Å, and refined in a riding mode with *U*_iso_(H) = 1.2*U*_eq_(C) and 1.5*U*_eq_(C,N).

### Crystal data

**Table:**

(C_7_H_10_N)_3_[SbCl_5_]Cl	*F*(000) = 1320
*M**_r_* = 658.93	*D*_x_ = 1.531 Mg m^−^^3^
Monoclinic, *P*2_1_/*c*	Mo *K*α radiation, λ = 0.71073 Å
Hall symbol: -P 2ybc	Cell parameters from 5029 reflections
*a* = 17.171 (3) Å	θ = 3.1–27.6°
*b* = 9.4065 (19) Å	µ = 1.54 mm^−^^1^
*c* = 20.958 (8) Å	*T* = 293 K
β = 122.36 (2)°	Block, colorless
*V* = 2859.4 (13) Å^3^	0.36 × 0.32 × 0.28 mm
*Z* = 4	

### Data collection

**Table:**

Rigaku SCXmini diffractometer	5029 independent reflections
Radiation source: fine-focus sealed tube	3776 reflections with *I* > 2σ(*I*)
graphite	*R*_int_ = 0.061
CCD_Profile_fitting scans	θ_max_ = 25.0°, θ_min_ = 3.1°
Absorption correction: multi-scan (*CrystalClear*; Rigaku, 2005)	*h* = −20→20
*T*_min_ = 0.566, *T*_max_ = 0.640	*k* = −11→11
23714 measured reflections	*l* = −24→24

### Refinement

**Table:**

Refinement on *F*^2^	Primary atom site location: structure-invariant direct methods
Least-squares matrix: full	Secondary atom site location: difference Fourier map
*R*[*F*^2^ > 2σ(*F*^2^)] = 0.045	Hydrogen site location: inferred from neighbouring sites
*wR*(*F*^2^) = 0.085	H-atom parameters constrained
*S* = 1.07	*w* = 1/[σ^2^(*F*_o_^2^) + (0.0238*P*)^2^ + 3.2828*P*] where *P* = (*F*_o_^2^ + 2*F*_c_^2^)/3
5029 reflections	(Δ/σ)_max_ = 0.001
286 parameters	Δρ_max_ = 0.45 e Å^−^^3^
0 restraints	Δρ_min_ = −0.57 e Å^−^^3^

### Special details

**Table:**

Geometry. All esds (except the esd in the dihedral angle between two l.s. planes) are estimated using the full covariance matrix. The cell esds are taken into account individually in the estimation of esds in distances, angles and torsion angles; correlations between esds in cell parameters are only used when they are defined by crystal symmetry. An approximate (isotropic) treatment of cell esds is used for estimating esds involving l.s. planes.
Refinement. Refinement of F^2^ against ALL reflections. The weighted R-factor wR and goodness of fit S are based on F^2^, conventional R-factors R are based on F, with F set to zero for negative F^2^. The threshold expression of F^2^ > 2sigma(F^2^) is used only for calculating R-factors(gt) etc. and is not relevant to the choice of reflections for refinement. R-factors based on F^2^ are statistically about twice as large as those based on F, and R- factors based on ALL data will be even larger.

### Fractional atomic coordinates and isotropic or equivalent isotropic displacement parameters (Å^2^)

**Table:**

	*x*	*y*	*z*	*U*_iso_*/*U*_eq_	
C1	0.5562 (4)	0.3197 (7)	0.3106 (4)	0.109 (2)	
H1A	0.5571	0.4166	0.2967	0.164*	
H1B	0.5821	0.3142	0.3640	0.164*	
H1C	0.4937	0.2861	0.2842	0.164*	
C6	0.7590 (3)	0.1338 (5)	0.3223 (3)	0.0463 (11)	
N1	0.8598 (2)	0.1246 (4)	0.3760 (2)	0.0598 (11)	
H1D	0.8881	0.1332	0.3510	0.090*	
H1E	0.8739	0.0409	0.3994	0.090*	
H1F	0.8781	0.1941	0.4099	0.090*	
C3	0.5726 (3)	0.1547 (5)	0.2232 (3)	0.0597 (14)	
H3	0.5093	0.1608	0.1890	0.072*	
C7	0.7080 (3)	0.2161 (5)	0.3400 (3)	0.0564 (13)	
H7	0.7370	0.2646	0.3858	0.068*	
C5	0.7193 (3)	0.0606 (5)	0.2556 (3)	0.0544 (13)	
H5	0.7547	0.0049	0.2439	0.065*	
C2	0.6120 (3)	0.2288 (5)	0.2900 (3)	0.0574 (14)	
C4	0.6246 (4)	0.0718 (6)	0.2059 (3)	0.0645 (15)	
H4	0.5961	0.0225	0.1604	0.077*	
C15	0.6589 (5)	0.6670 (8)	0.1612 (4)	0.109 (2)	
H15A	0.5931	0.6597	0.1358	0.163*	
H15B	0.6833	0.7087	0.2101	0.163*	
H15C	0.6848	0.5740	0.1668	0.163*	
N3	0.8982 (3)	0.8778 (5)	0.1259 (3)	0.0741 (13)	
H3A	0.9256	0.9322	0.1670	0.111*	
H3B	0.9028	0.9188	0.0898	0.111*	
H3C	0.9254	0.7931	0.1368	0.111*	
C18	0.7989 (4)	0.8600 (6)	0.0991 (3)	0.0604 (14)	
C16	0.6829 (4)	0.7576 (6)	0.1162 (3)	0.0653 (16)	
C17	0.7770 (4)	0.7709 (6)	0.1383 (3)	0.0669 (15)	
H17	0.8228	0.7192	0.1791	0.080*	
C19	0.7337 (4)	0.9369 (6)	0.0383 (3)	0.0748 (17)	
H19	0.7509	0.9967	0.0126	0.090*	
C20	0.6404 (5)	0.9249 (7)	0.0149 (4)	0.091 (2)	
H20	0.5948	0.9753	−0.0265	0.109*	
C21	0.6195 (4)	0.8376 (7)	0.0548 (4)	0.0789 (18)	
H21	0.5579	0.8309	0.0398	0.095*	
C8	0.6102 (4)	0.5583 (6)	−0.0837 (3)	0.092 (2)	
H8A	0.5445	0.5476	−0.1093	0.139*	
H8B	0.6301	0.5295	−0.1166	0.139*	
H8C	0.6265	0.6560	−0.0696	0.139*	
C13	0.7928 (3)	0.3667 (5)	0.0939 (3)	0.0463 (11)	
N2	0.8939 (2)	0.3478 (4)	0.1373 (2)	0.0596 (11)	
H2A	0.9108	0.2925	0.1771	0.089*	
H2B	0.9212	0.4321	0.1529	0.089*	
H2C	0.9107	0.3073	0.1080	0.089*	
C10	0.6068 (4)	0.3990 (6)	0.0117 (4)	0.0782 (17)	
H10	0.5429	0.4085	−0.0154	0.094*	
C12	0.7431 (4)	0.3015 (6)	0.1186 (3)	0.0657 (15)	
H12	0.7718	0.2477	0.1626	0.079*	
C9	0.6563 (4)	0.4669 (5)	−0.0139 (3)	0.0589 (14)	
C14	0.7512 (4)	0.4478 (5)	0.0293 (3)	0.0538 (13)	
H14	0.7875	0.4909	0.0141	0.065*	
C11	0.6489 (4)	0.3181 (7)	0.0760 (4)	0.0848 (19)	
H11	0.6131	0.2734	0.0911	0.102*	
Sb1	0.89514 (2)	0.62601 (3)	0.415711 (17)	0.04348 (11)	
Cl3	0.88469 (9)	0.44094 (13)	0.32367 (8)	0.0642 (4)	
Cl4	0.72983 (9)	0.63871 (17)	0.35705 (8)	0.0752 (4)	
Cl5	0.89748 (9)	0.80416 (12)	0.33449 (7)	0.0545 (3)	
Cl6	0.91678 (9)	0.85259 (14)	0.50888 (7)	0.0625 (4)	
Cl1	0.96680 (9)	0.10773 (12)	0.26801 (7)	0.0559 (3)	
Cl2	1.10143 (8)	0.63935 (13)	0.48357 (7)	0.0537 (3)	

### Atomic displacement parameters (Å^2^)

**Table:**

	*U*^11^	*U*^22^	*U*^33^	*U*^12^	*U*^13^	*U*^23^
C1	0.082 (5)	0.112 (6)	0.139 (7)	0.027 (4)	0.063 (5)	−0.020 (5)
C6	0.035 (2)	0.035 (2)	0.057 (3)	0.002 (2)	0.017 (2)	0.006 (2)
N1	0.046 (2)	0.044 (2)	0.070 (3)	−0.002 (2)	0.019 (2)	−0.007 (2)
C3	0.042 (3)	0.054 (3)	0.068 (4)	0.002 (3)	0.019 (3)	0.013 (3)
C7	0.052 (3)	0.050 (3)	0.058 (3)	−0.004 (2)	0.023 (3)	−0.016 (3)
C5	0.054 (3)	0.048 (3)	0.056 (3)	0.008 (2)	0.026 (3)	−0.006 (3)
C2	0.048 (3)	0.049 (3)	0.081 (4)	0.006 (2)	0.038 (3)	0.002 (3)
C4	0.056 (4)	0.064 (3)	0.051 (3)	0.002 (3)	0.014 (3)	−0.009 (3)
C15	0.105 (6)	0.131 (7)	0.092 (5)	−0.017 (5)	0.054 (5)	0.001 (5)
N3	0.060 (3)	0.077 (3)	0.091 (3)	−0.003 (3)	0.044 (3)	−0.005 (3)
C18	0.059 (3)	0.063 (3)	0.074 (4)	−0.014 (3)	0.045 (3)	−0.023 (3)
C16	0.077 (4)	0.073 (4)	0.067 (4)	−0.029 (3)	0.053 (4)	−0.028 (3)
C17	0.075 (4)	0.068 (4)	0.064 (4)	−0.005 (3)	0.041 (3)	−0.023 (3)
C19	0.061 (4)	0.076 (4)	0.089 (5)	−0.003 (3)	0.041 (4)	0.000 (4)
C20	0.073 (5)	0.098 (5)	0.105 (5)	−0.008 (4)	0.050 (4)	0.005 (4)
C21	0.057 (4)	0.099 (5)	0.077 (5)	−0.013 (4)	0.033 (4)	−0.025 (4)
C8	0.100 (5)	0.077 (4)	0.083 (5)	0.019 (4)	0.036 (4)	0.017 (4)
C13	0.039 (3)	0.039 (3)	0.054 (3)	−0.006 (2)	0.021 (2)	−0.011 (2)
N2	0.049 (3)	0.052 (3)	0.071 (3)	−0.004 (2)	0.028 (2)	−0.009 (2)
C10	0.048 (3)	0.083 (4)	0.086 (5)	0.006 (3)	0.025 (3)	0.005 (4)
C12	0.065 (4)	0.072 (4)	0.061 (4)	−0.003 (3)	0.034 (3)	0.011 (3)
C9	0.063 (4)	0.045 (3)	0.058 (3)	0.006 (3)	0.025 (3)	0.001 (3)
C14	0.062 (4)	0.040 (3)	0.065 (4)	−0.010 (2)	0.038 (3)	−0.003 (3)
C11	0.056 (4)	0.106 (5)	0.107 (5)	−0.003 (3)	0.054 (4)	0.025 (4)
Sb1	0.04486 (19)	0.03558 (17)	0.0501 (2)	0.00541 (15)	0.02546 (15)	0.00729 (15)
Cl3	0.0684 (9)	0.0456 (7)	0.0781 (10)	−0.0013 (6)	0.0388 (8)	−0.0083 (7)
Cl4	0.0484 (8)	0.0886 (11)	0.0848 (10)	0.0051 (8)	0.0331 (8)	0.0039 (9)
Cl5	0.0633 (8)	0.0436 (7)	0.0570 (8)	0.0114 (6)	0.0325 (7)	0.0160 (6)
Cl6	0.0567 (8)	0.0648 (9)	0.0676 (9)	0.0076 (7)	0.0343 (7)	−0.0057 (7)
Cl1	0.0608 (8)	0.0441 (7)	0.0609 (8)	0.0055 (6)	0.0312 (7)	0.0050 (6)
Cl2	0.0490 (7)	0.0505 (7)	0.0526 (7)	0.0027 (6)	0.0212 (6)	0.0055 (6)

### Geometric parameters (Å, °)

**Table:**

C1—C2	1.510 (7)	C17—H17	0.9300
C1—H1A	0.9600	C19—C20	1.406 (8)
C1—H1B	0.9600	C19—H19	0.9300
C1—H1C	0.9600	C20—C21	1.350 (8)
C6—C7	1.360 (6)	C20—H20	0.9300
C6—C5	1.369 (6)	C21—H21	0.9300
C6—N1	1.478 (5)	C8—C9	1.504 (7)
N1—H1D	0.8900	C8—H8A	0.9600
N1—H1E	0.8900	C8—H8B	0.9600
N1—H1F	0.8900	C8—H8C	0.9600
C3—C4	1.373 (7)	C13—C12	1.357 (6)
C3—C2	1.375 (7)	C13—C14	1.375 (6)
C3—H3	0.9300	C13—N2	1.478 (5)
C7—C2	1.408 (7)	N2—H2A	0.8900
C7—H7	0.9300	N2—H2B	0.8900
C5—C4	1.388 (6)	N2—H2C	0.8900
C5—H5	0.9300	C10—C11	1.368 (8)
C4—H4	0.9300	C10—C9	1.382 (7)
C15—C16	1.484 (8)	C10—H10	0.9300
C15—H15A	0.9600	C12—C11	1.376 (7)
C15—H15B	0.9600	C12—H12	0.9300
C15—H15C	0.9600	C9—C14	1.390 (7)
N3—C18	1.492 (6)	C14—H14	0.9300
N3—H3A	0.8900	C11—H11	0.9300
N3—H3B	0.8900	Sb1—Cl2	3.0319 (14)
N3—H3C	0.8900	Sb1—Cl3	2.5325 (14)
C18—C17	1.360 (7)	Sb1—Cl4	2.4182 (15)
C18—C19	1.369 (7)	Sb1—Cl5	2.4043 (13)
C16—C21	1.381 (8)	Sb1—Cl6	2.7779 (14)
C16—C17	1.428 (7)		
			
C2—C1—H1A	109.5	C16—C17—H17	120.4
C2—C1—H1B	109.5	C18—C19—C20	119.6 (6)
H1A—C1—H1B	109.5	C18—C19—H19	120.2
C2—C1—H1C	109.5	C20—C19—H19	120.2
H1A—C1—H1C	109.5	C21—C20—C19	117.6 (6)
H1B—C1—H1C	109.5	C21—C20—H20	121.2
C7—C6—C5	121.5 (4)	C19—C20—H20	121.2
C7—C6—N1	119.3 (4)	C20—C21—C16	124.7 (6)
C5—C6—N1	119.2 (4)	C20—C21—H21	117.6
C6—N1—H1D	109.5	C16—C21—H21	117.6
C6—N1—H1E	109.5	C9—C8—H8A	109.5
H1D—N1—H1E	109.5	C9—C8—H8B	109.5
C6—N1—H1F	109.5	H8A—C8—H8B	109.5
H1D—N1—H1F	109.5	C9—C8—H8C	109.5
H1E—N1—H1F	109.5	H8A—C8—H8C	109.5
C4—C3—C2	121.4 (5)	H8B—C8—H8C	109.5
C4—C3—H3	119.3	C12—C13—C14	121.7 (5)
C2—C3—H3	119.3	C12—C13—N2	118.8 (5)
C6—C7—C2	120.9 (5)	C14—C13—N2	119.5 (4)
C6—C7—H7	119.5	C13—N2—H2A	109.5
C2—C7—H7	119.5	C13—N2—H2B	109.5
C6—C5—C4	118.2 (5)	H2A—N2—H2B	109.5
C6—C5—H5	120.9	C13—N2—H2C	109.5
C4—C5—H5	120.9	H2A—N2—H2C	109.5
C3—C2—C7	117.2 (5)	H2B—N2—H2C	109.5
C3—C2—C1	122.4 (5)	C11—C10—C9	121.9 (5)
C7—C2—C1	120.4 (5)	C11—C10—H10	119.1
C3—C4—C5	120.7 (5)	C9—C10—H10	119.1
C3—C4—H4	119.6	C13—C12—C11	117.5 (5)
C5—C4—H4	119.6	C13—C12—H12	121.3
C16—C15—H15A	109.5	C11—C12—H12	121.3
C16—C15—H15B	109.5	C10—C9—C14	116.0 (5)
H15A—C15—H15B	109.5	C10—C9—C8	121.9 (5)
C16—C15—H15C	109.5	C14—C9—C8	122.0 (5)
H15A—C15—H15C	109.5	C13—C14—C9	121.5 (5)
H15B—C15—H15C	109.5	C13—C14—H14	119.3
C18—N3—H3A	109.5	C9—C14—H14	119.3
C18—N3—H3B	109.5	C10—C11—C12	121.4 (5)
H3A—N3—H3B	109.5	C10—C11—H11	119.3
C18—N3—H3C	109.5	C12—C11—H11	119.3
H3A—N3—H3C	109.5	Cl5—Sb1—Cl4	93.76 (5)
H3B—N3—H3C	109.5	Cl5—Sb1—Cl3	87.77 (5)
C17—C18—C19	122.3 (5)	Cl4—Sb1—Cl3	93.52 (5)
C17—C18—N3	118.2 (5)	Cl5—Sb1—Cl6	85.20 (5)
C19—C18—N3	119.4 (5)	Cl4—Sb1—Cl6	89.91 (5)
C21—C16—C17	116.5 (5)	Cl3—Sb1—Cl6	172.36 (4)
C21—C16—C15	123.8 (6)	Cl5—Sb1—Cl2	81.10 (4)
C17—C16—C15	119.6 (6)	Cl4—Sb1—Cl2	174.39 (4)
C18—C17—C16	119.2 (6)	Cl3—Sb1—Cl2	88.54 (4)
C18—C17—H17	120.4	Cl6—Sb1—Cl2	87.44 (4)
			
C5—C6—C7—C2	0.0 (8)	N3—C18—C19—C20	−177.3 (5)
N1—C6—C7—C2	179.3 (4)	C18—C19—C20—C21	0.7 (9)
C7—C6—C5—C4	−0.3 (7)	C19—C20—C21—C16	−1.0 (10)
N1—C6—C5—C4	−179.6 (4)	C17—C16—C21—C20	0.5 (9)
C4—C3—C2—C7	−0.1 (8)	C15—C16—C21—C20	177.3 (6)
C4—C3—C2—C1	−179.5 (5)	C14—C13—C12—C11	0.7 (8)
C6—C7—C2—C3	0.2 (7)	N2—C13—C12—C11	−178.4 (5)
C6—C7—C2—C1	179.5 (5)	C11—C10—C9—C14	0.1 (9)
C2—C3—C4—C5	−0.2 (8)	C11—C10—C9—C8	−178.7 (6)
C6—C5—C4—C3	0.3 (8)	C12—C13—C14—C9	−0.1 (7)
C19—C18—C17—C16	−0.7 (8)	N2—C13—C14—C9	179.1 (4)
N3—C18—C17—C16	176.8 (4)	C10—C9—C14—C13	−0.4 (7)
C21—C16—C17—C18	0.3 (7)	C8—C9—C14—C13	178.5 (5)
C15—C16—C17—C18	−176.6 (5)	C9—C10—C11—C12	0.6 (10)
C17—C18—C19—C20	0.2 (9)	C13—C12—C11—C10	−1.0 (9)

### Hydrogen-bond geometry (Å, °)

**Table:**

*D*—H···*A*	*D*—H	H···*A*	*D*···*A*	*D*—H···*A*
N1—H1D···Cl1	0.89	2.72	3.602 (4)	169
N1—H1E···Cl6^i^	0.89	2.67	3.513 (4)	158
N1—H1F···Cl2^ii^	0.89	2.59	3.455 (4)	163
N2—H2A···Cl1	0.89	2.37	3.240 (4)	166
N2—H2B···Cl1^iii^	0.89	2.40	3.249 (4)	160
N2—H2C···Cl2^iv^	0.89	2.41	3.238 (4)	155
N3—H3A···Cl1^v^	0.89	2.47	3.344 (5)	167
N3—H3B···Cl2^iii^	0.89	2.56	3.366 (5)	151
N3—H3C···Cl1^iii^	0.89	2.55	3.355 (5)	151

Symmetry codes: (i) *x*, *y*−1, *z*; (ii) −*x*+2, −*y*+1, −*z*+1; (iii) −*x*+2, *y*+1/2, −*z*+1/2; (iv) −*x*+2, *y*−1/2, −*z*+1/2; (v) *x*, *y*+1, *z*.
